# The prevalence of metabolic syndrome in cardiovascular patients in Iran: a systematic review and meta-analysis

**DOI:** 10.1186/s13098-020-00605-4

**Published:** 2020-11-03

**Authors:** Nader Salari, Peyman Kaikhosro Doulatyari, Alireza Daneshkhah, Aliakbar Vaisi-Raygani, Rostam Jalali, Parnian kord Jamshidi, Alireza Abdi, Masoud Mohammadi, Mohsen Kazeminia

**Affiliations:** 1 Department of Biostatistics, School of Health, Kermanshah University of Medical Sciences, Kermanshah, Iran; 2 Cardiovascular research center, Kermanshah University of Medical Sciences, Kermanshah, Iran; 3 School of Computing, Electronics and Maths, Coventry University, London, United Kingdom; 4 Department of Nursing, School of Nursing and Midwifery, Kermanshah University of Medical Sciences, Kermanshah, Iran; 5 Department of Obstetrics and Gynecology, School of Medicine, Kermanshah University of Medical Sciences, Kermanshah, Iran; 6 Student Research Committee, Kermanshah University of Medical Sciences, Kermanshah, Iran

**Keywords:** Metabolic syndrome, cardiovascular, prevalence, meta-analysis, Iran

## Abstract

**Background:**

Cardiovascular disease is the cause of more than 50% of mortalities globally, and this rate has grown by 8.6% since the 60 s. One of the risk factors associated with cardiovascular disease and its resulting mortality rate is the metabolic syndrome. Different studies have reported inconsistent rates for the metabolic syndrome. However, no comprehensive study has been conducted to combine the results of existing studies. Thus, the present study was performed with the aim of determining the prevalence of metabolic syndrome among cardiovascular patients in Iran through a systematic review and meta-analysis.

**Method:**

: In this review study, the Scientific Information Database, Google Scholar, Science Direct, Scopus, PubMed, and Web of Science (ISI), databases were searched from January 2005 and until May 2020, to identify and extract related articles. To conduct the analysis, a random effects model was used, and the heterogeneity of the studies was examined using the I^2^ index. Data analysis was performed within Comprehensive Meta-Analysis (version 2) software.

**Results:**

The prevalence of metabolic syndrome in cardiovascular patients in Iran in the 27 papers examined with a sample size of 44,735 patients was 34.2% (95% CI: 26.8–42.6%). A sensitivity analysis was performed to ensure the stability of the results, these results show that by omitting the prevalence from each study, the overall prevalence (34.2%) does not change significantly. the highest prevalence of metabolic syndrome in studies conducted in the period between 2015 and 2020, and this was reported as 55.3 (95% CI: 47.9–62.3) and the highest prevalence of metabolic syndrome in studies conducted in the methods of diagnosis IDF, and the rate was reported as 48 (95% CI: 36.5–59.8). based on meta-regression as the year of research increased, the prevalence of metabolic syndrome in cardiovascular patients in Iran also increased. However, with the increase in sample size, this prevalence decreased (p < 0.05).

**Conclusions:**

The results of this study indicate that metabolic syndrome is high in cardiovascular patients in Iran. Accordingly, by understanding its etiology and supervision at all levels, suitable solutions could be offered by providing feedback to hospitals.

## Background

Cardiovascular disease generally refers to conditions that involve narrowed or blocked blood vessels that can lead to a heart attack, chest pain (angina) or stroke. Other heart conditions, such as those that affect your heart’s muscle, valves or rhythm, also are considered forms of heart disease [1–4].

Cardiovascular disease is the cause of more than 50% of mortalities in world, and this figure has grown by 8.6% since the 60 s [1, 2]. The mortality caused by cardiovascular disease in the US is higher than that resulting from cancer, accidents, and diabetes [3]. This disease affects the elderly more than the other age groups, such that 83% of those who die because of cardiovascular disease are above 65 years of age [2]. The World Health Organization (WHO) has predicted that, over the upcoming two decades, the mortality caused by cardiovascular disease may grow to 137% and 120% in men and women, respectively [4].

One of the risk factors associated with cardiovascular disease and its resulting mortality rate is metabolic syndrome [5, 6]. The individuals suffering metabolic syndrome are 3–5 times more likely to develop cardiovascular disease and die as a result, compared to non-affected individuals [7, 8]. Metabolic syndrome was initially referred to as hypertension, diabetes, and gout according to Reaven study in 1988; Gans study in 2006 also reported insulin resistance as the central characteristic of this disorder and called it the X syndrome [9, 10]. Metabolic syndrome increases the risk of cardiovascular disease in patients at any level of LDL [6–9].

The definitions proposed for metabolic syndrome are different in terms of execution as well as the boundary of their diagnosis. Nevertheless, abdominal obesity, hypertension, elevated glucose levels, and dyslipidemia exist in all definitions of the syndrome. Two definitions were previously proposed by the Adult Treatment Panel (ATPIII) [10, 11]. Recently, the International Diabetes Federation has presented a new definition for metabolic syndrome. In this definition, the waist circumference, which is different for various races, has been used as a major factor [12].

On the other hand, the dangerous role of metabolic syndrome in the incidence of coronary heart diseases (CHD) is increasing [13], which has developed into a major health problem in human societies [14]. Patients with cardiovascular disease need to pay close attention to their risk of catching other diseases, given the dangerous side effects of the disease, many of which are fatal. The most significant of these diseases is metabolic syndrome, which can double the effects of cardiovascular disease and increase its incidence [13–15].

CHD has been known as one of the major causes of mortality [15]. Nevertheless, the information around the relationship between metabolic syndrome based on different definitions and CHD [16], especially in developing countries is still unclear [17]. There is sparse information available about the relationship between each of the definitions of metabolic syndrome and the risk of incidence of CHD. For instance, for Europeans [18, 19], as well as American Hindus [20], metabolic syndrome based on the WHO and ATPIII definitions, has widely predicted the incidence of CHD.

In Iran, a high prevalence of metabolic syndrome has been reported [21]. The prevalence of metabolic syndrome in patients with cardiovascular disease in Isfahan was 1.9% [23], in Tehran 36.5% [24], and in the city of Arak, this rate was reported 7.5% [25].

Considering the effect of different factors on the prevalence of metabolic syndrome in cardiovascular patients and the lack of general statistics in this regard across Iran, we intended to perform our review study on the existing studies, and approximate the overall prevalence using suitable meta-analysis techniques. The goal was to achieve general statistics about the prevalence of metabolic syndrome in cardiovascular patients in Iran.

## Method

The study population in this study are cardiovascular patients, and, we were looking for the prevalence of metabolic syndrome reported as an outcome.

In this systematic review and meta-analysis study, the prevalence of metabolic syndrome in cardiovascular patients in Iran was examined based on the studies published between January 2005 and May 2020. For this purpose, the papers published in national and international databases of Scientific Information Database, Google scholar, Science Direct, Scopus, PubMed, and Web of Science were searched through English or the Persian equivalents of the following keywords: prevalence, metabolic syndrome, cardiovascular, and Iran.

Cross-sectional studies were included, yet review papers, case-controls, cohort, and interventional studies were excluded from the list of articles. Duplicate publications and multiple publications from the same population were removed from the list of articles that had been prepared within the EndNote (version X7, for Windows, Thomson Reuters) reference management software.

### Study selection

Initially, all papers that assessed the prevalence of metabolic syndrome in cardiovascular patients in Iran were collected. Then, the studies were examined, based on the inclusion and exclusion criteria. The exclusion criteria were irrelevant topics, case reports, interventional studies, duplicate studies, unclear methods, and lack of access to the full text of the paper. In order to reduce the bias, the search of the papers was performed independently by two reviewers (for the search process, examination of titles and abstracts, full-text assessment, data extraction, and Quality assessment); in case of disagreement between the two reviewers, that paper was examined by the head of the group. A total of 35 studies were included in the third stage, i.e. quality evaluation.

### Quality evaluation of studies

The quality of the papers was assessed based on the selected and relevant items of a 22-item STROBE checklist. The fields of the checklist were study design, background and review of texts, place and time of study, outcomes, inclusion criteria, sample size, and statistical analysis. The papers that had fulfilled 6–7 items of the criteria were considered as high-quality papers, while those not satisfying two or 3–5 of the seven items were considered as low or medium-quality papers (methodological quality) articles respectively Accordingly, the maximum quality score that could be obtained from the checklist was 32; papers with a score of less than 14 were considered as low quality, and were therefore excluded from the study. [22]. In the present study, 27 papers were included in the systematic review and meta-analysis as medium or high quality articles, while eight studies which were assessed as low quality were removed.

### Data extraction

All of the final papers introduced into the meta-analysis process were prepared for extraction using a different pre-prepared checklist. The checklist included: title of paper, name of first author, year of publication, place of study, sample size, prevalence of metabolic syndrome, and methodology.

### Statistical analysis

Since the prevalence has a binomial distribution, the variance of prevalence was calculated using a binomial distribution variance formula. Additionally, to combine the value of prevalence across different studies, weighted average was used. To assess the heterogeneity of the selected studies, the I^2^ index test was used. To examine the publication bias, considering the large sample size of the studies included, the Begg’s (Begg and Mazumdar) test at the significance level of 0.1 was used, and the corresponding Funnel plots were constructed. To inspect the extent of effect of every individual study on the final outcome, sensitivity analysis test was utilized. Data analysis was performed within the Comprehensive Meta-Analysis (version 2) software.

## Results

**Fig. 1:**
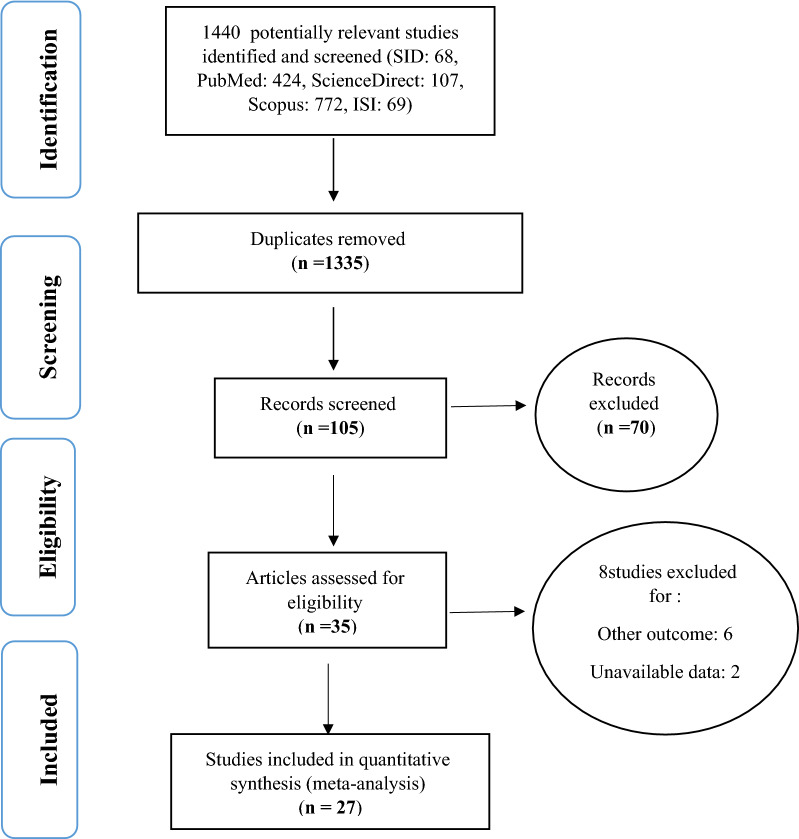
Flow diagram of study selection

In this study, all of the studies conducted on the prevalence of metabolic syndrome in cardiovascular patients in Iran, published between January 2005 and until May 2020, were examined in accordance with the Preferred Reporting Items for Systematic Reviews and Meta-Analyses (PRISMA) guidelines. In the preliminary research of the SID, PubMed, ScienceDirect, Scopus and ISI databases, 1440 papers were included. Articles satisfying the initial inclusion criteria were 105, after the elimination of 1335 duplicate articles. With detailed assessments of the articles and exclusion of further 8 studies (6 with unrelated outcomes, and 2 with unavailable data), finally, 27 articles entered the meta-analysis process (Fig. 1).

**Fig. 2:**
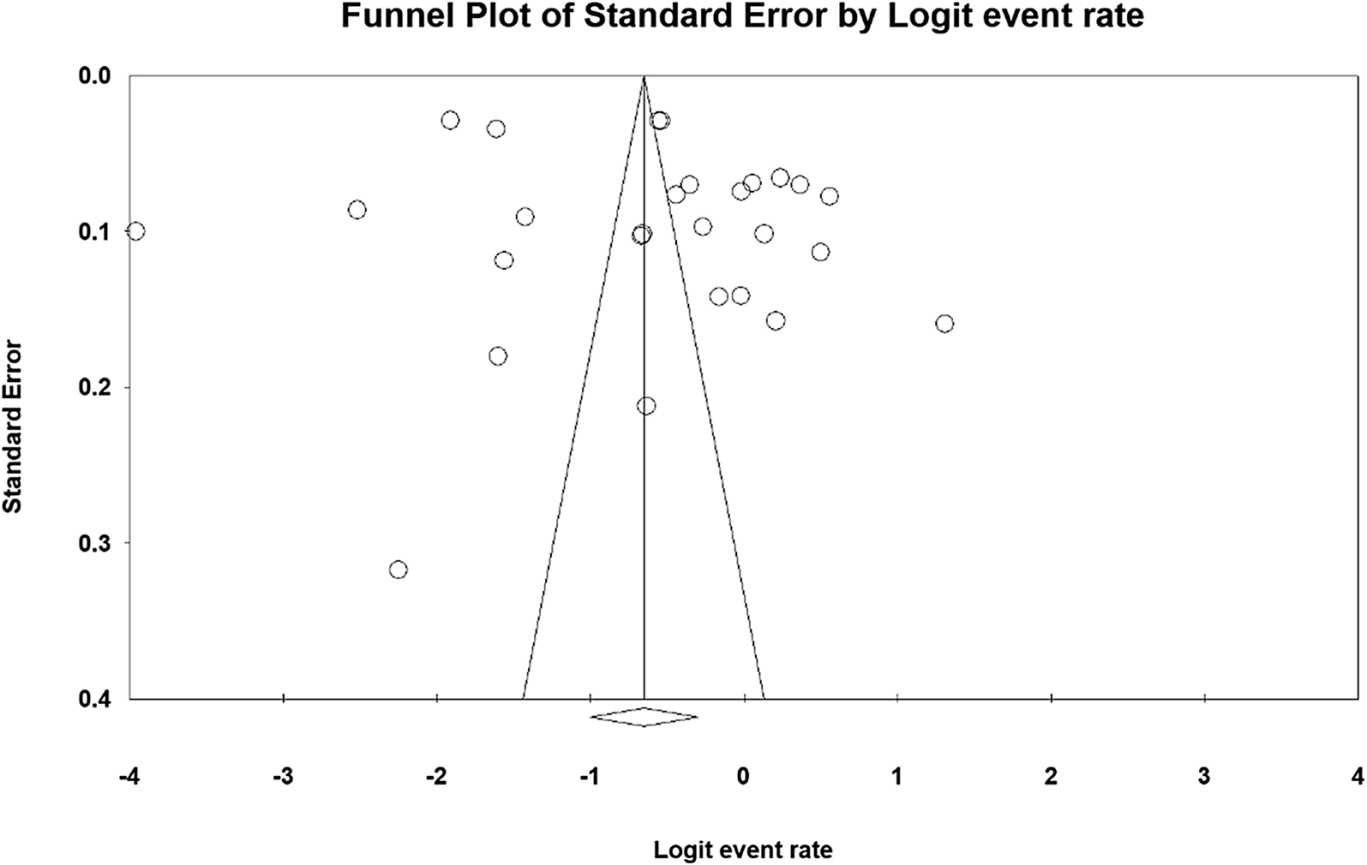
Funnel plot of the results related to the prevalence of metabolic syndrome cardiovascular patients in Iran

The publication bias using the funnel plot and Begg and Mazumdar test at the significance level of 0.1, highlighting no publication bias in the present study (P = 0.297) (Fig. 2).

Based on the results obtained from the test (I^2^:99) and considering the heterogeneity of the selected studies, a random effects model was used for combining the studies and for the overall estimation of the prevalence.

**Fig. 3:**
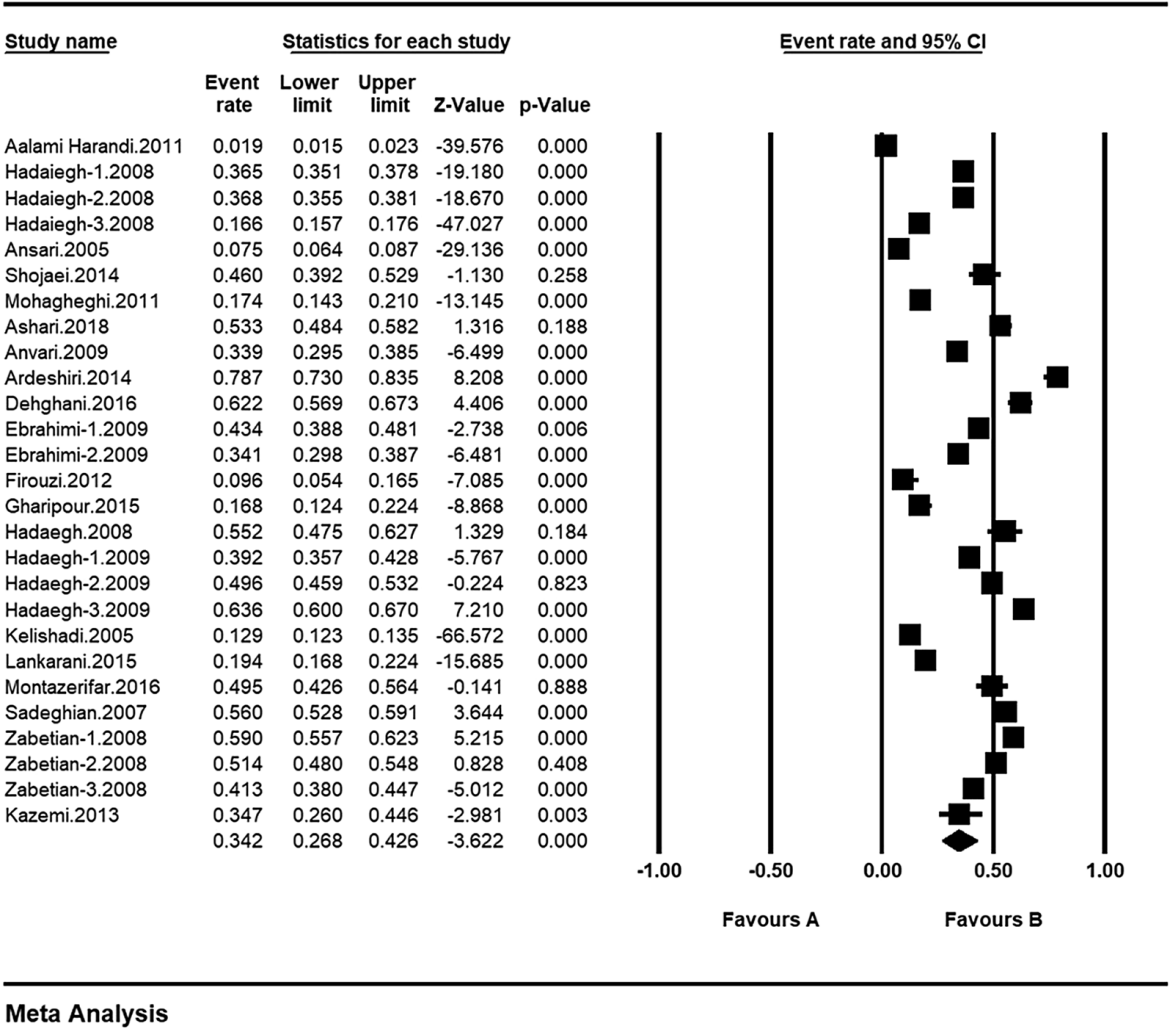
Prevalence of metabolic syndrome in cardiovascular patients and confidence interval 95% in Iran

The total number of participants selected in the meta-analysis was 4473. The characteristics of the studies included in the systematic review are shown in Table 1. The maximum and minimum prevalence of metabolic syndrome in cardiovascular patients were related to the studies by Anvari et al. 33.9% [29] and Aalami Harandi et al. 1.9% [23]. Based on the results of the study, the overall prevalence of metabolic syndrome in cardiovascular patients in Iran is 34.2% (95% CI: 26.8–42.6%) (Fig. 3).

**Table 1:** Characteristic of included studies Prevalence of metabolic syndrome in cardiovascular patients

Author, year, [Reference]	Methods of diagnosis	City	Sample size	Prevalence %	Quality
Aalami Harandi, 2011, [23]	ATPIII	Isfahan	5431	1.9	Medium
Hadaiegh-1, 2008, [24]	ATPIII	Tehran	5141	36.5	High
Hadaiegh-2, 2008, [24]	IDF	Tehran	5141	36.8	High
Hadaiegh-3, 2008, [24]	WHO	Tehran	5141	16.6	High
Ansari, 2005, [25]	ATPIII	Arak	1939	7.5	High
Shojaei, 2014, [26]	ATPIII	Saghez	200	46.0	High
Mohagheghi, 2011, [27]	WHO	Tehran	495	17.4	High
Ashari, 2018, [28]	ATPIII	Hamedan	390	53.3	High
Anvari, 2009, [29]	ATPIII	Tehran	422	33.9	High
Ardeshiri, 2014, [30]	ATPIII	Tehran	235	78.7	Medium
Dehghani, 2016, [31]	ATPIII	Urmia	331	62.2	Medium
Ebrahimi-1, 2009, [32]	IDF	Mashhad	431	43.4	High
Ebrahimi-2, 2009, [32]	ATPIII	Mashhad	431	34.1	High
Firouzi, 2012, [33]	ATPIII	Tehran	115	9.6	High
Gharipour, 2015, [34]	ATPIII	Isfahan	220	16.8	Medium
Hadaegh, 2008, [35]	ATPIII	Tehran	163	55.2	High
Hadaegh-1, 2009, [36]	ATPIII	Tehran	720	39.2	High
Hadaegh-2, 2009, [36]	WHO	Tehran	720	49.6	High
Hadaegh-3, 2009, [36]	IDF	Tehran	720	63.6	High
Kelishadi, 2005, [37]	ATPIII	Isfahan	10,814	12.9	Medium
Lankarani, 2015, [38]	ATPIII	Shiraz	777	19.4	High
Montazerifar, 2016, [39]	ATPIII	Zahedan	200	49.5	High
Sadeghian, 2007, [40]	ATPIII	Tehran	940	56.0	High
Zabetian-1, 2008, [41]	ATPIII	Tehran	840	59.0	High
Zabetian-2, 2008, [41]	IDF	Tehran	840	51.4	High
Zabetian-3, 2008, [41]	WHO	Tehran	840	41.3	High
Kazemi, 2013, [42]	ATPIII	Birjand	98	34.7	High

### Sensitivity analysis

**Fig. 4:**
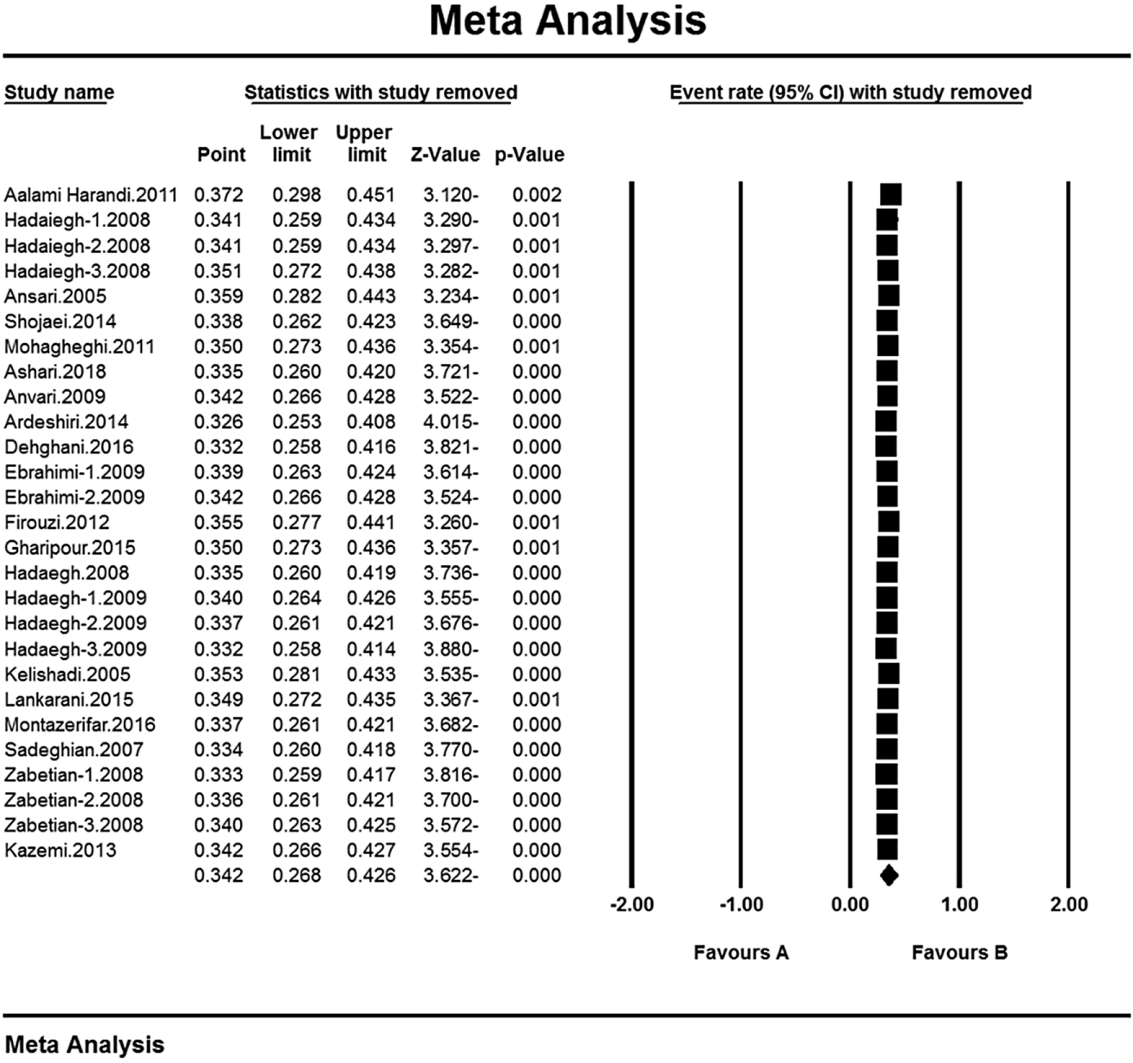
Results of sensitivity analysis

A sensitivity analysis was perfumed to ensure the stability of the results, after removing each study results and observing no change (Fig. 4).

**Fig. 5:**
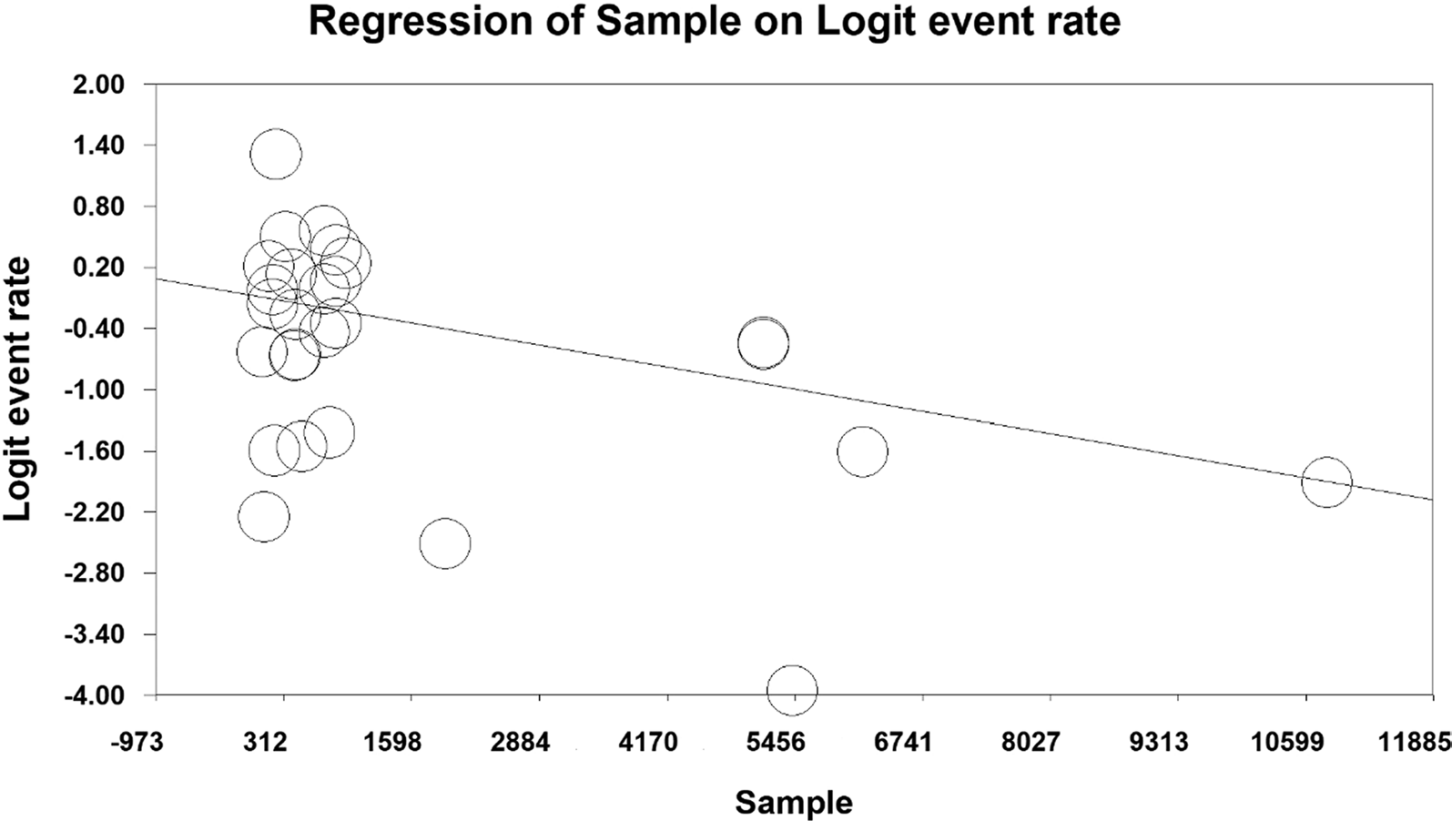
Meta-regression of the relationship between the sample size and prevalence of metabolic syndrome in cardiovascular patients in Iran

**Fig. 6:**
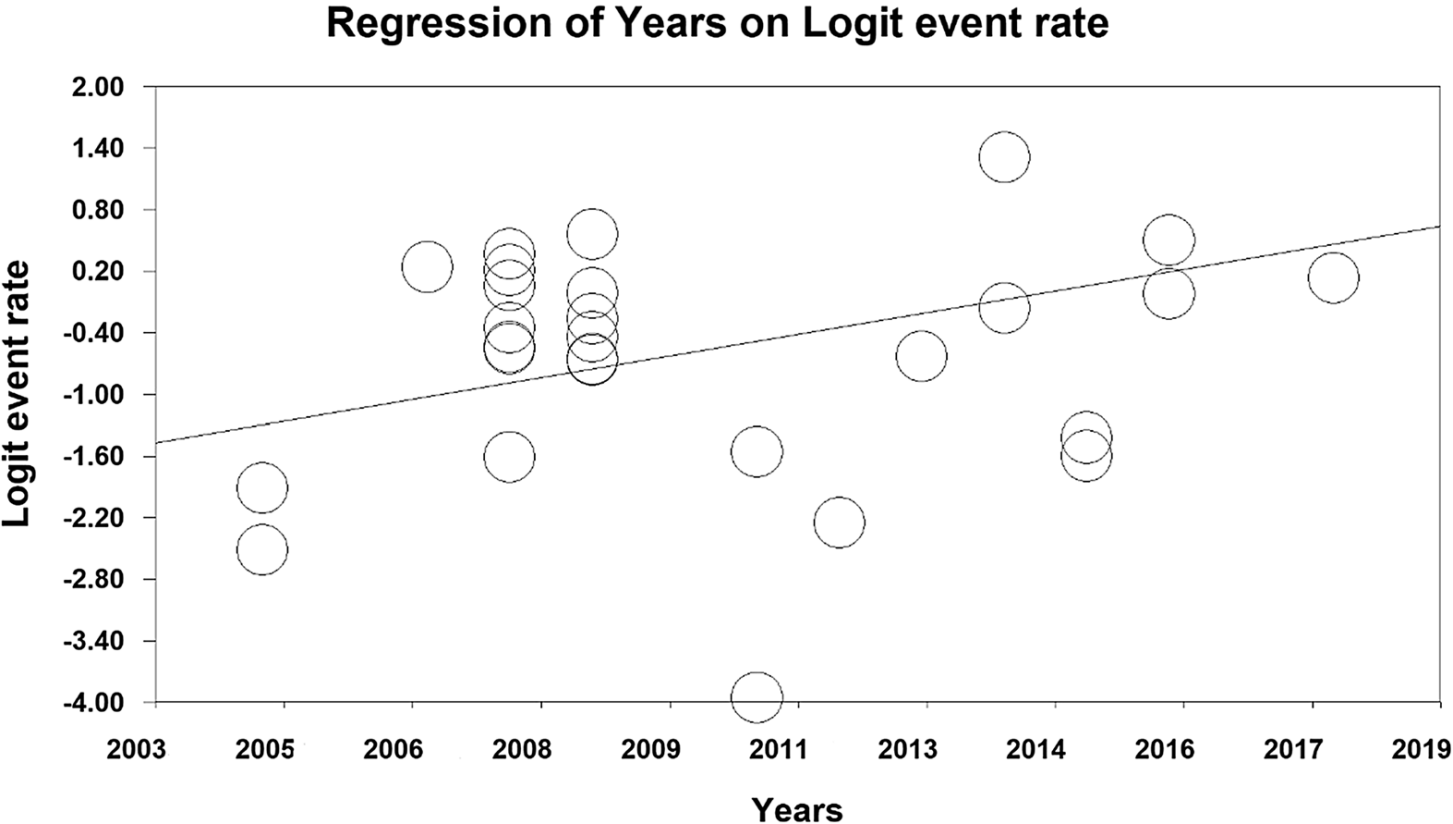
Meta-regression of the relationship between the year of the study and prevalence of metabolic syndrome in cardiovascular patients in Iran

The relationship between the year of conducting studies (P = 0.000), sample size (P = 0.000) and the prevalence of metabolic syndrome in Iranian cardiovascular patients was investigated using the meta-regression analysis. A significant difference was observed between the prevalence of metabolic syndrome and each of the two mentioned variables. As the year of research increased, the prevalence of metabolic syndrome in cardiovascular patients in Iran also increased. However, with the increase in sample size, this prevalence decreased (Figs. 5 and 6).

### Subgroup analysis based on year of study and methods of diagnosis of metabolic syndrome

Considering the results of Table 2 and the subgroup analysis based on year of study, the highest prevalence of metabolic syndrome in studies conducted in the period between 2015 to 2020, and this was reported as 55.3 (95% CI: 47.9–62.3)’ considering the subgroup analysis and based on the methods of diagnosis of metabolic syndrome, the highest prevalence of metabolic syndrome in studies conducted in the methods of diagnosis IDF, and the rate was reported as 48 (95% CI: 36.5–59.8).

**Table 2:** Subgroup analysis on the years of study and Methods of diagnosis of metabolic syndrome

	Time range	Number of articles	Sample size	I^2^	Begg and Mazumdar	Prevalence (95% CI)
Year of studies	2005–2010	16	36,243	99.6	0.102	37.6 (95% CI: 28.5–47.6)
	2011–2015	8	7571	99.3	0.379	21.7 (95% CI: 13.2–47.1)
	2016–2020	3	921	79.3	0.783	55.3 (95% CI: 47.9–62.3)
Methods of diagnosis of metabolic syndrome	ATPIII	19	29,739	99.5	0.528	30.9 (95% CI: 21.3–42.3)
	WHO	3	6701	99.4	0.296	35.7 (95% CI: 18.1–58.2)
	IDF	5	7295	98.3	0.806	48 (95% CI: 36.5–59.8)

## Discussion

The present research was conducted with the aim of determining the prevalence of metabolic syndrome in cardiovascular patients in Iran. In the present systematic review, a total of 27 selected studies with a sample size of 43,735 subjects were investigated. For meta-analysis, using a random effects model, the total prevalence of metabolic syndrome in cardiovascular patients is found as 34.2% (95% CI: 26.8–42.6%). The high prevalence of metabolic syndrome in cardiovascular patients in Iran, as obtained in the present study, confirms the strong relationship between this syndrome and cardiovascular diseases. Based on the results of the present study, the maximum prevalence of metabolic syndrome in cardiovascular patients was associated with cardiac ischemic disease patients.

The prevalence was obtained as 73.2% by Rashidi among patients with type II diabetes [44]. Furthermore, in a research conducted by Aguilar et al. in 2003–2012 in the US, the total prevalence of metabolic syndrome was reported as 33% [45]; the figure obtained in Iran is higher than other countries which requires relevant attention.

The results of our study can follow prospective studies revealing that metabolic syndrome based on ATPIII definition and whether it is a predictor for CHD after modifying the variables of age, serum LDL, smoking, and history of premature CHD [43–48]. In NHANES III cross-sectional study on the American population above 50 years of age, metabolic syndrome based on ATPIII, in the presence of its components, did not present better prediction of CHD [49–55]. In contrast, in another study, metabolic syndrome based on ATPIII (and not WHO) was known as an independent risk factor for CHD after modifying the components of syndrome and cardiovascular risk factors [48]. Eventually, Ford, in a review study, found that metabolic syndrome based on ATPIII and WHO only plays an average role in predicting CHD (with relative risk of 1.7–1.9%) [56].

With regards to the effect of year of study and the prevalence of metabolic syndrome in cardiovascular patients in Iran, it was observed that this prevalence has an ascending trend among Iranian patients. Thus, interventions should be put in place. These interventions should result in a change of lifestyle, regularly controlling blood sugar and lipid levels of patients to prevent the disease and its associated complications. In addition to this, as metabolic syndrome is preventable in the first place, and, and to prevent or control complications, cardiovascular patients should receive complete training on the disease and the ways for preventing its complications. Moreover, it is important to note that through early diagnosis of complications, the disease can be treated and controlled.

When assessing the findings of this study, the current limitations should also be mentioned. The most important limitation of this study was that its papers were cross-sectional; prospective studies are required to investigate the relationship between different definitions of metabolic syndrome and CHD. Secondly, considering the high prevalence of diabetes [57] and obesity [58–60] in our Country, it seems that the reported prevalence of metabolic syndrome and CHD has been underestimated in this study [23–42]. One of the major strengths of this work was the usage of a large sample size of patients with cardiovascular disease in Iran, thus enhancing the generalizability of our findings.

Considering the high prevalence of metabolic syndrome in cardiovascular patients in Iran, it is suggested that physicians should pay more attention to the symptoms of this disease. Additionally, training should be offered through relevant platforms and media to raise the awareness of individuals, to promote early diagnosis of the condition. Furthermore, considering the prevalence of metabolic syndrome in cardiovascular patients in other parts of the world, further studies should be conducted in order to identify the prevalence of this condition worldwide.

## Limitations

One of the most important limitations of the present study is the lack of access to the full text of some studies due to their low quality and high heterogeneity of studies.

## Conclusions

The results of this study suggest that the prevalence of metabolic syndrome in cardiovascular patients in Iran is high. Accordingly, to improve this situation, to find the etiology, and for supervision at all levels, suitable solutions should be offered by giving feedback to hospitals.

## Data Availability

Datasets are available through the corresponding author upon reasonable request.

## Abbreviations

SID: Scientific Information Database.
WHO: World Health Organization.
ATPIII: Adult Treatment Panel III.
IDF: International Diabetic Federation.
STROBE: Strengthening the Reporting of Observational Studies in Epidemiology.
LDL: Low-Density Lipoprotein.
NHANES III: The Third National Health and Nutrition Examination Survey.
CHD: Coronary Heart Diseases.
PRISMA: Preferred Reporting Items for Systematic Reviews and Meta-Analysis.
